# Learnings From the Collaborative Practices of Elite Sports Doubles Players: A Scoping Review

**DOI:** 10.7759/cureus.84787

**Published:** 2025-05-25

**Authors:** Katie Walker, Edson Filho, Jenny Rudolph, Michael Meguerdichian, Tricia Yusaf, Kimberly Campbell-Taylor, Maryam Asoodar

**Affiliations:** 1 School of Health Professions Education, Maastricht University, Maastricht, NLD; 2 Wheelock College of Education and Human Development, Boston University, Boston, USA; 3 Center for Medical Simulation, Harvard University, Cambridge, USA; 4 The Simulation Center, New York City Health + Hospitals, New York, USA

**Keywords:** collaborative practices, elite sports teams, expert healthcare dyads, expert performance, expert sports doubles, healthcare teams

## Abstract

When healthcare teams fail to communicate clearly and follow through on shared plans, patient outcomes and clinician well-being are compromised. Although substantial research has explored the factors that contribute to effective team performance, far less is known about what supports or hinders the performance of the foundational unit within teams: the dyad. The effectiveness of dyads, such as the emergency nurse/emergency physician or midwife/obstetrician pair, is a critical determinant of clinician well-being, patient safety, and quality of care.

Drawing inspiration from the coordinated efforts of elite tennis doubles teams, this review examines the collaborative behaviors of high-performing sporting duos and considers how those insights may inform dyadic functioning in healthcare. What can the commitment to practice and the high-stakes mindset of athletic partnerships teach us about what is required of clinical dyads? Using the frameworks of distributed cognition and relational coordination, we analyze how these athletes effectively distribute cognitive workload across individuals and their environments to optimize performance.

A scoping review was conducted using Maastricht University’s LibSearch, which includes PsycINFO, MEDLINE, the Education Resources Information Center, and Web of Science. Searches were carried out between March 13, 2020, and July 4, 2021. Following the Preferred Reporting Items for Systematic reviews and Meta-Analyses guidelines for scoping reviews, 14 studies met the inclusion criteria.

From this review, five key categories of collaborative practices used by elite sports doubles teams to enhance teamwork emerged. These include performance evaluation, frequent and structured practice, ongoing feedback from coaches, developing a unique team culture that promotes a shared mindset, and recruitment strategies that prioritize prior experience and preexisting familiarity between partners.

Based on these findings, the authors propose that adopting similar strategies in healthcare, particularly cultivating a distinct team culture grounded in shared cognitive frameworks, and prioritizing dyadic familiarity during recruitment, could meaningfully improve both dyadic and broader team performance in clinical environments.

## Introduction and background

Rationale

There is a growing call for enhanced collaboration in acute care settings to reduce persistent errors, highlighting the need to upskill healthcare professionals as they navigate increasingly dynamic environments [1,2]. Traditionally, exceptional doctors have been revered as singular figures, celebrated for their individual clinical expertise. However, the journey toward excellent practice is increasingly recognized as a collective endeavor. A critical step in advancing collaborative practice is developing a nuanced understanding of the attributes and behaviors that underpin expert team performance.

While the literature on team dynamics is extensive, comparatively little attention has been paid to the dyad: the smallest functional unit of collaboration [3], comprising two individuals working closely toward a shared goal. In healthcare, dyads are found across various clinical environments, including surgeon-anesthetist pairs in the operating room, intensivist-critical care nurse teams in the ICU, and midwife-obstetrician pairs in maternity care. These partnerships play a pivotal role in shaping team effectiveness and patient outcomes.

Collaborative practices, a necessity for these partnerships, refer to the specific behaviors, strategies, and communication methods teams use to coordinate their actions, build trust, and achieve shared goals [4,5]. These practices include explicit actions, such as structured handovers and shared decision-making, and implicit processes like nonverbal communication and situational awareness.

To explore these collaborative practices more deeply, we drew on two key theoretical frameworks: relational coordination (RC) [6] and distributed cognition (DC) [7].

Relational Coordination

RC focuses on achieving task integration through mutually reinforcing communication and relationships. It highlights the importance of shared goals, shared knowledge, and mutual respect in enabling high-quality performance.

Distributed Cognition

Hutchins realized that, until the mid-1990s, cognitive science had largely focused on the individual agent as the unit of analysis. However, in most human endeavors, outcomes result from interactions between multiple experts and technical systems. Proponents of DC theory identify six key factors that promote robust team performance: 1) frequent direction or guidance, 2) status reporting, 3) alert reporting, 4) goal sharing, 5) problem solving, and 6) frequent explanation. Hutchins found that when these elements are addressed, team performance improves.

Analyzing the collaborative practices of elite sports doubles teams through the frameworks of RC and DC helps to identify the strengths and gaps in the collaborative capabilities of healthcare dyads. These frameworks were established prior to the commencement of our scoping review and informed both our search strategy and analysis. Insights from these theories have allowed us to unpack the complexities of collaboration and better understand how excellence is achieved in fields outside of healthcare.

Salas et al. [8] define an expert team as one composed of interdependent members, each possessing advanced knowledge, skills, and experience relevant to their tasks. Such teams are capable of adapting, coordinating, and cooperating effectively to produce consistent, high-level performance. We hypothesize that these expert teams achieve excellence through deliberate collaborative practices designed to improve team functioning and develop expertise over time. In this review, we focus specifically on the dyad or doubles team as the unit of analysis, rather than on individual expert performance.

Building on this, Halldorsson et al. [9], in reference to sports teams, introduce the concept of sociality, which emphasizes simultaneous performance through concurrent awareness of oneself, one’s abilities, and the abilities of others. Halldorsson et al. argue that this provides a strong foundation for systematic research into teamwork, particularly in sports where successful collaboration directly translates into measurable outcomes. This makes sports an ideal context for analyzing the culture, traditions, and social fundamentals of team success [4]. These findings offer valuable inspiration for healthcare dyads, where, as with doubles players, success can be measured through tangible outcomes such as improved clinical results and reduced errors.

Objectives

This scoping review explores the breadth of the available literature, summarizes the evidence, and identifies directions for future research, with the overarching aim of providing a comprehensive evidence map on collaborative practices [10,11].

The three main research questions guiding this review are as follows: How are the collaborative practices of elite doubles players and teams conceptualized and studied? What specific insights can the collaborative practices of elite sports doubles players provide to expert healthcare dyads and teams? How can these insights inform and guide future research on the collaborative practices of healthcare dyads?

## Review

Methods

Protocol and Registration

Our protocol was drafted using the Preferred Reporting Items for Systematic reviews and Meta-Analyses extension for scoping review (PRISMA-ScR) tool. The final protocol was registered prospectively with the Open Science Framework on https://osf.io/hnu76/.

Eligibility Criteria

Of the array of literature reviews available, we chose a scoping review methodology as it provides an overview of how a topic is being conceptualized and studied in a field or domain. It can provide the state-of-the-science information on a topic. The eligibility criteria for the review are described in Table 1.

**Table 1:** Inclusion and exclusion criteria

Criterion	Inclusion	Exclusion
Date	2016 to June 30, 2021	Before 2016 and after June 30, 2021
Exposure of interest	Elite sports teams/doubles players analysis and collaborative practices	Individual learning practices
Language	English	All other languages
Participants	Elite sports team players	Exclude all single-person skill acquisition and novice learning
Peer review	Peer-reviewed literature and non-peer-reviewed	None
Objective measures	Measuring the number and type of collaborative practices elite sports teams use	None
Reported outcomes	Using objective measures and self-reported data	None
Setting	Match play and practice sessions	None
Type of publication	Original studies, commentaries, reviews and editorials, and position papers	None

We included studies of elite players in any sports team focusing on the collaborative practices that drove expert performance. We assessed each study to ensure that collaborative expertise was studied, not individual skill acquisition. We reviewed this problem specifically in sports practice and match play settings. A preliminary search of MEDLINE, the Cochrane Database of Systematic Reviews, and Joanna Briggs Institute Evidence Synthesis was conducted, and no current or underway systematic reviews or scoping reviews on the topic were identified. Settings in all countries were included, and there were no racial, ethnic, or gender-based exclusions. Only manuscripts from January 2016 to June 30, 2021, were included, and only those written in English were reviewed.

Information Sources

The databases we searched comprised Maastricht University LibSearch, including PsycINFO, MEDLINE, Education Resources Information Center, and Web of Science. The references of all included manuscripts were searched, and the relevant articles were included. The searches were conducted between March 13, 2020, and July 4, 2021.

Search

The text words contained in the titles and abstracts of relevant articles and index terms were used. Table 2 presents a full search strategy for the American Psychological Association PsycINFO.

**Table 2:** Search strategy: PsycINFO

No.	Searches	Results
S1	(Elite sports) and (doubles performance)	0
S2	(“Sports doubles”) AND (Communication)	6
S3	(“sports doubles”) and (“Expert performance”)	0
S4	(“sports doubles”) and (practice)	0
S5	(“sports dyads”) AND (“perform*”)	18
S6	(“Sports doubles”) AND (Coordination”)	2
S7	(“dyads” OR “athlet* dyads” OR “sport dyads” OR “sport doubles”) AND (“communicat*” OR “perform*” OR “coordinat*” OR “expect*” OR “practice”)	2,470
S8	(“dyads” OR “athlet* dyads” OR “sport dyads” OR “sport doubles”) AND (“communicat*” OR “perform*” OR “coordinat*” OR “expect*” OR “practice”) AND (“cognitive load”)	1
S9	(“dyad” OR “athlet* dyads” OR “sport dyads” OR “sport doubles”) AND (“communicat*” OR “perform*” OR “coordinat*” OR “expect*” OR “practice” OR “cognit*”)	1,162
S10	(“elite dyad*” OR “expert sports team*” OR “expert double*” OR “elite double*player*”) AND (Learn* OR practic* OR educat* OR “practice” OR communic* OR coordinat* or perform*)	887
S11	(“sports dyad*” OR “sports team*” OR “doubles player” OR “tennis players”) AND (“Learn*” OR “practic*” OR “educat*” OR “communic*” OR “coordinat*” or “perform*”)	2,469

The librarians at a research-intensive university in the Netherlands were advisors to the research team and played a key role in assisting them in refining the search terms.

The final search string was: (“sports dyad*” OR “sports team*” OR “doubles player” OR “tennis players”) AND (“Learn*” OR “practic*” OR “educat*” OR “communic*” OR “coordinat*” or “perform*”).

Numerous sports require two people to work closely together, such as badminton, pickleball, sailing, and rowing. For practical purposes, only tennis players were included in the string.

Selection of Sources of Evidence

In this study, we mapped the literature on the collaborative practices of sporting dyads to better understand how this topic has been characterized and studied within the sports domain. This research focused specifically on the rehearsals and practices employed by experienced teams of two to directly improve collaboration and team performance. For empirical studies, each citation was characterized by the year of publication, location of the study by continent, study setting (match play, practice, or both), number of participants, type of sport, research design, and the learning practices identified. Review articles were characterized by the type of review, the population studied, and the main ideas and recommendations presented. Commentaries were categorized based on the recommendations or main ideas provided.

Following the search, all identified citations were collated and uploaded into EndNote X9 (June 2019, Clarivate Analytics, PA, USA), and duplicates were removed. Any disagreements that arose between reviewers during the selection process were resolved through discussion with an additional reviewer. A random sample of 25 titles and abstracts was selected and reviewed by a team of four researchers. Once 75% agreement was achieved, the team proceeded with screening the remaining records. A total of 987 records and abstracts were reviewed, resulting in 68 full-text articles selected for further assessment, with reasons for exclusion clearly documented. Two researchers independently reviewed all records and abstracts against the inclusion criteria, and any discrepancies were resolved by a third reviewer. The same process was followed for full-text manuscript review, with justifications for exclusion explicitly stated. This resulted in a final inclusion of 14 full-text manuscripts.

**Figure 1:**
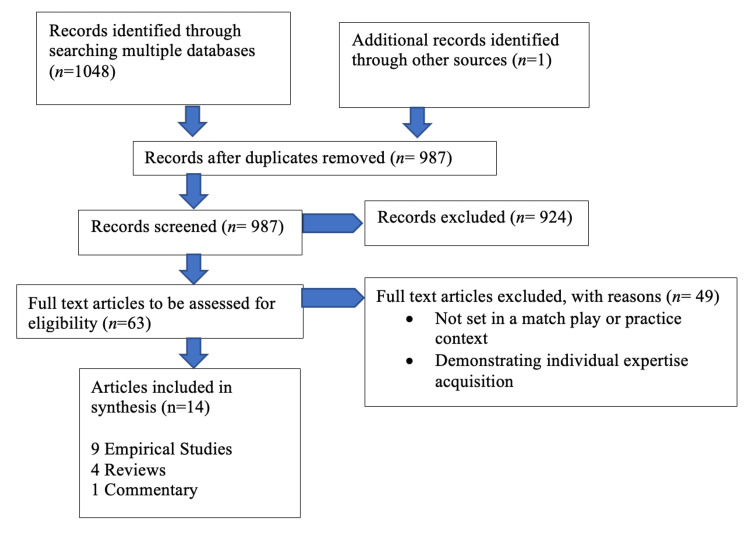
Flowchart on the selection of sources of evidence

The results of the search and study selection process are presented in full using the PRISMA-ScR flow diagram (see Figure 1).

Data Charting Process

Four authors independently charted the data from each eligible article using a SurveyMonkey (San Mateo, CA) tool. Any disagreements were resolved through a virtual call discussion between the two reviewers and further determination by a third reviewer. The draft data extraction tool was modified and revised as necessary during the process of extracting data from each included evidence source (Table 3).

**Table 3:** Data extraction tool

Data extraction tool
Scoping review details
1. Name of reviewer
2. Date of review
Evidence source details and characteristics
1. Citation details: author/s, title, journal, volume, issue, pages, year of publication, and location
2. Study setting
3. Number of participants enrolled in the study
4. Type of sport
Details/results extracted from the source of evidence
1. Which learning practices were identified?
2. How were learning practices conducted?
3. How was learning measured?
4. Was there a specific guide for future learning?
5. What was the research design?
6. Was there agreement or controversy among the authors about the efficacy of the learning practices?
7. Were specific learning practices suggested to optimize future learning?
8. Were there gaps identified in the uptake of effective learning practices?

Synthesis of Results

We summarized the empirical studies by type of settings, populations, study design, and the number of sports professionals participating. We created a table of the different types of collaborative practices identified and where they were applied, in match play, a practice setting, or both. We grouped the 19 collaborative practices into five categories. The research team met to determine category names and which collaborative practice would be included in each group. Due to the heterogeneity of study designs and the exploratory nature of this review, no meta-analysis or statistical pooling of data was conducted.

Results

Selection of Sources of Evidence

Fourteen studies discussed collaborative practices of elite sports teams in practice and match play settings. Of the empirical studies, the majority were from Europe (six), with two from North America, and one from Australasia.

Characteristics of Sources of Evidence

Of the 14 manuscripts included in the synthesis, nine were empirical studies, four were reviews, and one was a commentary. Collaborative practices used by expert sports teams include 19 unique collaborative practices found in the literature (Table 4).

**Table 4:** Sports teams' collaborative practices MP: match play; PS: practice setting

Observable practices or examples	MP	PS	MP and PS	Total
Video review with individual and group analysis	0	0	1	1
Recruiting players who have practiced and played together previously	1	0	0	1
Each athlete taking leadership responsibilities	1	0	0	1
Cultivating a special team shared mentality	1	0	0	1
Shared knowledge of each players skills and personal styles	1	0	0	1
Team goal setting with all players	0	0	1	1
Each player participates in individual goal setting			1	1
Prebrief and debrief each time you play	0	0	1	1
Using simulations to practice difficult situations	0	0	1	1
Receiving frequent feedback from coaches			1	1
Implementing a team charter including milestones and how to achieve them	0	0	1	1
Coaches inspiring and motivating team members each time you play	0	0	1	1
Cultivating team identity and togetherness based on a selfless culture	0	0	1	1
Exposing the team to challenging and unexpected training situations	0	0	1	1
Practicing enjoyment and keeping a positive outlook during stressful times in play	0	0	1	1
Practicing nonverbal communication	0	1	0	1
Provision of supportive coaching behaviors	0	1	0	1
Practicing mental toughness techniques, self-belief, and confidence training	0	1	0	1
Developing a team regulatory system based on ownership and responsibility	0	0	1	1
Total	4	3	12	19

From the literature, most practices were used in both practice and match play. This is significant when we think of healthcare dyads, as determining the setting for expert collaborative practice may affect access to practice. For example, sometimes it may be easier to practice in the clinical setting, and at other times, it may be better to practice in a simulation or training center. There were four practices: 1) recruiting players who have practiced and played together previously, 2) educating all players in leadership responsibilities, 3) cultivating a special team shared mentality, and 4) sharing knowledge of each player's skills and personal styles. Additionally, there were three practices only used in match play: 1) utilizing nonverbal communication, 2) supportive coaching behaviors and mental toughness techniques, and 3) self-belief and confidence training, which were only deployed in the practice setting. We found that most manuscripts on teamwork in sports teams were empirical studies, which included case studies, surveys, questionnaires, interviews, and observational studies. The nine empirical studies examined did not rate the efficacy of one collaborative practice over another or demonstrate any consistency on when or how the practice is applied. In most manuscripts, the efficacy of the practice was measured; however, there were no comparative studies on the efficacy of one practice over another or practices used in combination. This means it is still unknown which practices or combinations of practices work most effectively for each aspect of performance (e.g., routine building) and are less effective for other aspects of performance (e.g., decision-making). One study [12] focused on the efficacy of leadership training for each team member. Fransen et al. say that when leadership skills are learned by each team member, the team becomes characterized by high levels of team confidence and strong task and social cohesion. This social cohesion or familiarity may assist in building expert collaborative skills.

Many of the publications we reviewed were not specific about the practices deployed and did not unearth a shared understanding of teamwork, collaborative measurement, and measurement of team performance. In sports teams, constellations of practices were generally used; however, in each manuscript, the constellation of practices was different. Variability in terminology and definitions certainly hampers a clear understanding of what works, for whom, and under what circumstances. This can be problematic, however, when the aim is to understand how a field approaches a research topic, variability can also offer helpful contrasts to analyze the various approaches and concepts researchers have used.

We introduced the frameworks of RC and DC and have interpreted our findings on collaborative practices in relation to the theoretical frameworks in the following way. When considering the 19 practices identified, we found that elements of both RC and DC could be addressed through clustering the learning practices into the five broader topic areas: 1) evaluate performance, 2) frequent practice, 3) frequent feedback, 4) specific recruitment strategy, and 5) create unique team culture. These are broad categories, and further research is needed to understand the specifics of each category.

Synthesis of Results

In most empirical studies, more than one collaborative practice was adopted. All publications examined collaborative practices of teams that were larger than two participants, apart from Weinberg et al [13], who examined the stability of mental toughness in tennis teams. While most manuscripts reported improved team behaviors from implementing identified collaborative practices, only a few studies incorporated direct measures of doubles and team performance. There was no direct evidence of combinations of collaborative practices that were reproducible (transferable to different sporting groups) that improved performance. Of the quantitative studies, there was one each of survey and analysis, questionnaire, cross-sectional, and nonrandomized controlled intervention. Of the qualitative studies, two were case studies, one was an interview, one was a retrospective analysis, and one was an ethnography. Most of the research was conducted in both practice and match play settings.

Discussion

Summary of Evidence

In this discussion, we will describe key insights derived from the review and contextualize these insights in terms of existing evidence. We have also made recommendations for future research.

Our work broadens the current understanding of how collaborative practices used by elite sports doubles players are conceptualized and studied, highlighting the two unique categories of practices that differ from those of healthcare teams and that may influence performance. We have categorized these as "creating a unique team culture" and "deploying a specific recruitment strategy." Creating a unique team culture included practices that create a shared team mentality, creating a team charter and team regulatory system, practices that promote a shared team identity, and practices that promote a positive outlook. The specific recruitment strategy these teams deploy is recruiting players who have previously played together. All these practices promote familiarity, which is a key success factor in high-performing teams [3]. Familiarity is complicated in healthcare teams due to the temporality of members. There are practices that can be adopted to speed up familiarity in temporal healthcare teams.

Reflexivity

Our multidisciplinary research team included selected individuals as researchers from interprofessional backgrounds, including sports psychology, medical education researchers, behavioral psychology, nurses, and physicians.

One member of the research team has a career in human performance and optimization of elite sports teams, at both the individual and group levels. Two members of the team are Professors in Medical Education and have done research on expertise and expertise development. One researcher is a professor of behavioral science and a lifelong athlete. All authors have made substantive intellectual contributions to the development of this scoping review. During this process, our perspectives were both challenged and confirmed by our findings.

Findings from Walker et al.’s scoping review [14] of the collaborative practices of expert Healthcare Dyads and teams found five constellations of practices that were most frequently used. The authors of this sports collaborative practices review also found five patterns. In both sports and healthcare, three groupings, evaluate performance, frequent practice, and frequent feedback, were similar. There was a divergence with the other two groupings. In healthcare, there were just-in-time aids and studying ideal examples, whereas in elite sports teams, there were the sociocultural elements of creating a unique team culture and applying a specific recruitment strategy. Many questions stem from these findings. The authors are wondering if the practices that appeared to be similar between healthcare and sports are indeed identical. Do they aim for the same outcomes, and are they approached in the same or similar ways in both domains? Moreover, are there completely different angles to the challenge of making dyads work well within sports teams that are not yet used in healthcare?

There is a lack of data regarding the interdependence of collaborative practices that promote expert performance of elite doubles players and teams in match play or practice settings and only a few studies incorporated direct measures of doubles and team performance. There was no direct evidence that combinations of collaborative practices to improve performance are reproducible.

The distinctive elements mentioned previously of creating a unique team culture and applying a specific recruitment strategy may guide future research efforts as we further explore team excellence. The authors agree that this is not straightforward, as there are distinct differences between teams in sports and teams in healthcare, e.g., for sports teams, “excellence” is relatively easy to measure (their ranking in competitions, percentage of matches they win, etc.). Sports teams are more stable than the temporality of teams in healthcare. Healthcare teams are more diverse, with the inclusion of multiple professions and ages, which is another point of difference from sports teams.

**Figure 2:**
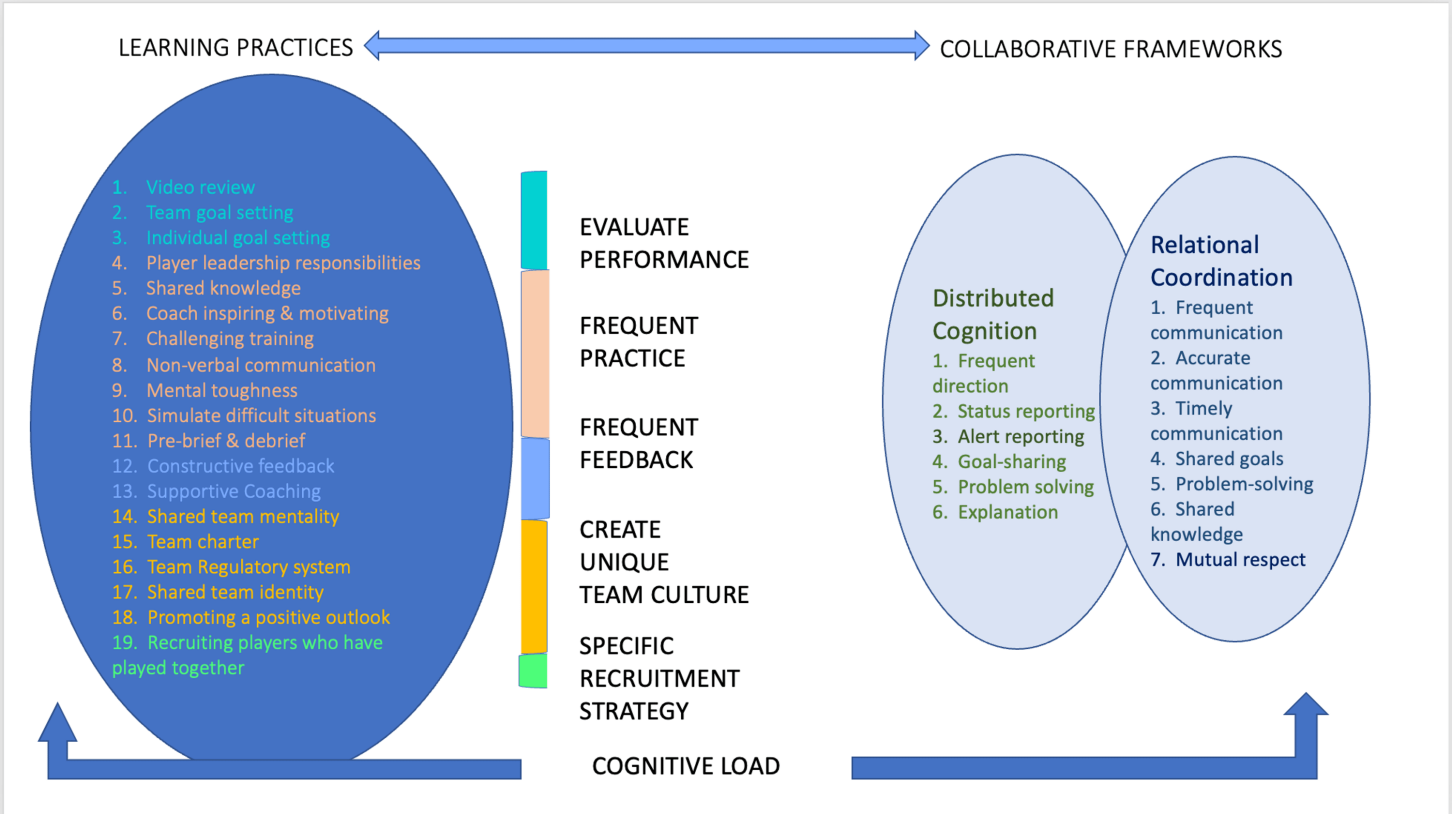
Collaborative practices of sports teams

Collaborative expertise is a complex and multifaceted phenomenon, and a thorough understanding of its individual components and how they interact remains an area for further exploration. Figure 2 shows the collaborative practices of sports teams, demonstrating different categories and the elements within.

Although most of the studies included in our review focused on teams larger than dyads, Santos et al. [15] refer to the idea that interpersonal coordination, or collaboration, unfolds at different levels or scales such as dyads, subgroups, and teams. Santos et al. reference that player/player interactions are the basic unit of analysis for studying interpersonal coordination in team sports.

We surmise that, as suggested by Dalal et al. [16], preassembly shared work experience can possibly help to decrease workload, freeing up processing resources that can be used for improved RC. Evidence from the reviewed studies highlights how prebriefing and debriefing foster DC, enabling doubles players to build shared mental models and coordinate more effectively.

In line with efforts to structure deliberate practice and improve knowledge retention in clinical settings, Koenig et al. [17] propose a spaced repetition model to optimize plastic surgery education, which may offer insights for structured cognitive training in healthcare dyads.

Future Research Recommendations for Healthcare Teams

Further research is recommended to map the outcomes of applying identified collaborative practices to improve team performance, specifically in understanding how the identified collaborative practices have been applied in sports teams.

While it is clear that valuable lessons can be drawn from the expertise of elite sports professionals, the specific components of their collaborative expertise remain underexplored. The aim of this scoping review was to examine how the collaborative practices of elite doubles and team sports have been conceptualized and studied and to offer recommendations for future research. One promising avenue for advancing our understanding of high-performing teams may involve directly engaging elite doubles players and exploring how they developed their expertise and the learning practices that contributed to their performance.

Currently, the evidence base is insufficient to define what constitutes effective collaborative practice or to inform the design of targeted interventions. Furthermore, there is a lack of clarity regarding how collaborative behaviors should be evaluated and measured in terms of their effectiveness. Theoretical frameworks such as RC and DC may offer useful lenses through which to examine and enhance collaboration within doubles teams. These frameworks can also inform the identification of best practices that may be transferable to healthcare dyads. This highlights a clear need for high-quality research to unpack the collaborative routines of elite doubles players and how these practices are enacted, adapted, and potentially translated into clinical contexts.

Limitations

This scoping review has several limitations. Due to the scarcity of research specifically focused on doubles collaborations, we broadened our inclusion criteria to encompass studies involving larger teams. While initially a limitation, this expansion also revealed a potential strength: existing research suggests that the performance of larger teams often depends on their weakest dyad [3]. This insight reinforces the importance of examining dyadic interactions within larger team contexts and suggests that understanding dyad-level functioning may be crucial for improving overall team performance.

Another limitation relates to our decision to use dyads as the primary unit of analysis rather than individuals. Given the limited body of research treating duos as a distinct analytical unit, this approach posed challenges but also contributes to a growing call within the literature to focus on "bottleneck pairs," those whose interactions critically influence team dynamics and outcomes [3]. We are pleased to contribute to this emerging area of study.

A further limitation stems from the search strategy employed. We did not include broader search terms such as “duos” or “pairs,” which may have captured additional relevant literature. Additionally, we limited the sports-related search strand to “tennis doubles,” anticipating it would yield the most pertinent results. This may have led to excluding studies from other relevant sports or domains. However, to address this, we conducted a comprehensive snowball search of reference lists from all included studies, which revealed further relevant work.

Despite these limitations, the review represents a meaningful step forward in understanding collaborative practices in elite sports partnerships. It highlights both gaps in the existing research and promising directions for future investigation. In particular, this review offers early insights into the dynamics of elite doubles teams, suggests valuable parallels for healthcare team performance, and underscores the need for more targeted research in this area.

## Conclusions

This study offers valuable insights into the collaborative dynamics of expert doubles in elite sports, highlighting critical relational and strategic practices that contribute to high performance. While the contexts of sport and healthcare differ, our findings suggest that healthcare dyads can meaningfully enhance their collaborative effectiveness by adopting practices demonstrated in elite sports teams. Specifically, fostering a unique team culture through the development of a shared team mentality, creating a team charter, establishing clear team regulatory systems, and promoting a positive, resilient outlook can strengthen psychological safety and team cohesion. Additionally, adopting targeted recruitment strategies-where feasible-to prioritize individuals with prior experience and established familiarity may further improve team performance and efficiency.

The strength of these conclusions is supported by the consistency of collaborative practices observed across high-performing sports teams, where RC and trust underpinned their success. These findings align with established theoretical frameworks, including RC and DC, underscoring their applicability to healthcare team dynamics.

To translate these insights into practice, healthcare organizations can begin by facilitating structured team-building interventions focused on shared goals, mutual accountability, and psychological safety. Introducing team charters and explicit discussion of collaborative expectations within healthcare dyads, the smallest team, can help solidify shared mental models. Where possible, consideration should be given to rostering or hiring strategies that leverage existing professional relationships to enhance familiarity and trust.

Recognizing the inherent differences between sports and healthcare environments, further research is recommended to explore how these practices can be effectively adapted and implemented in clinical settings. Longitudinal studies examining the impact of these interventions on team performance and patient outcomes will be crucial in refining and validating these strategies for healthcare teams.
